# First person – Camille Lacarrière-Keïta

**DOI:** 10.1242/dmm.052770

**Published:** 2025-12-29

## Abstract

First Person is a series of interviews with the first authors of a selection of papers published in Disease Models & Mechanisms, helping researchers promote themselves alongside their papers. Camille Lacarrière-Keïta is first author on ‘ Autophagy inhibition in intestinal stem cells favors enteroendocrine cell differentiation through Stat92E activity’, published in DMM. Camille conducted the research described in this article while a PhD student in Professor Steve Jean's lab at Université de Sherbrooke, Sherbrooke, Canada. She is now a postdoc researcher in the lab of Dr Edward A. Fon and Dr Ziv Gan-Or at McGill University, Ville-Marie, Canada, investigating GCase lysosomal trafficking and activity in dopaminergic neurons harbouring a reduced expression of lysosomal Parkinson's disease-risk genes.


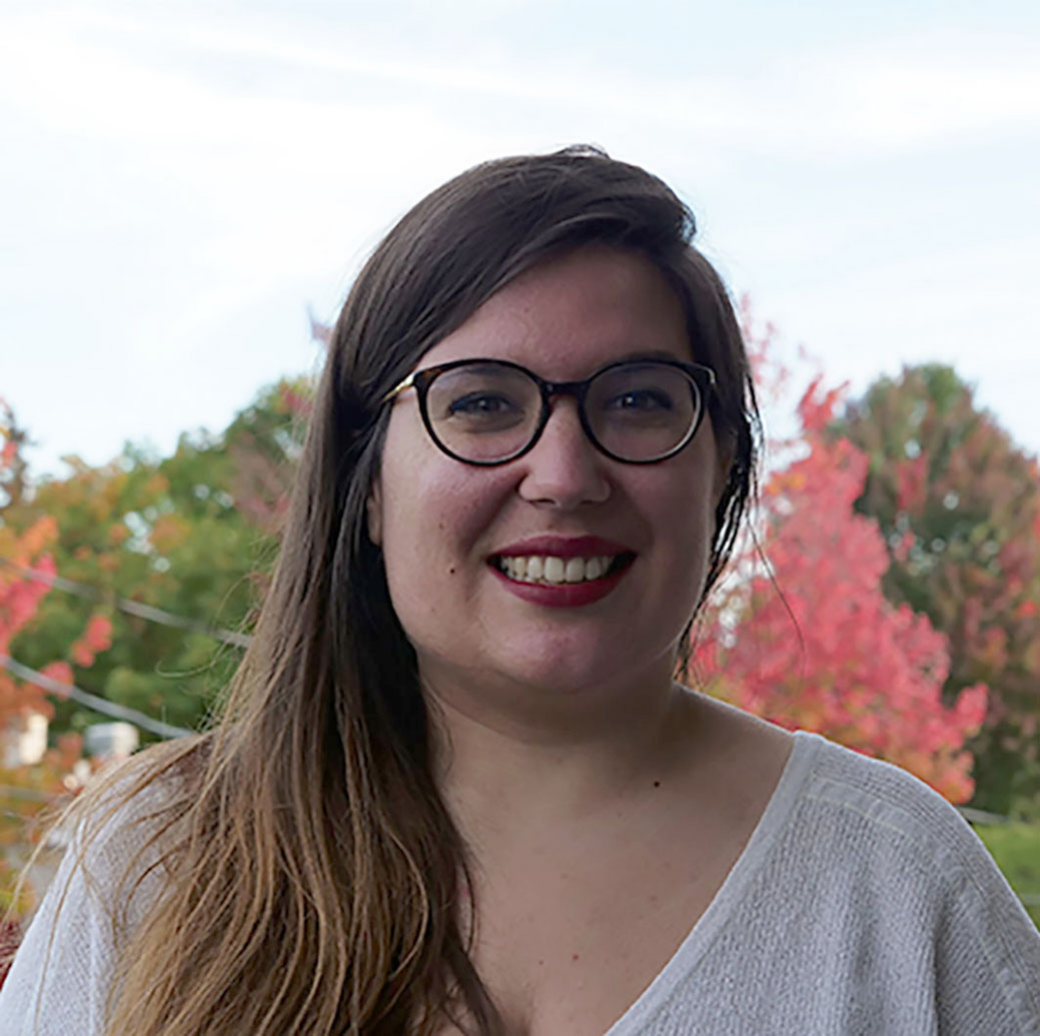


**Camille Lacarrière-Keïta**

**Who or what inspired you to become a scientist?**

After earning a diploma as a laboratory technician in biotechnology, I joined Dr Maryelle Tropis's laboratory at Toulouse University in France, first as a volunteer intern and later during my master's program. Dr Tropis was the first person who genuinely believed in my potential to become a scientist in academia. She showed me that research can also be creative and deeply enjoyable. She taught me to see hypotheses as puzzles waiting to be solved, each piece contributing to a clearer understanding of the bigger scientific picture. Her confidence in me and her passion for research are what inspired me to pursue a career in science.

**What is the main question or challenge in disease biology you are addressing in this paper? How did you go about investigating your question or challenge?**

Genetic alterations in autophagy regulators have been linked to inflammatory bowel diseases (IBDs). Patients with intestinal defects harbour an increased number of damaged epithelial cells, which should be replaced by new differentiated cells derived from intestinal stem cells. We wanted to determine whether inducing dysfunctional autophagy specifically in intestinal stem cells would be sufficient to disrupt gut organization in adult *Drosophila*, under basal conditions. To investigate this hypothesis, we knocked down autophagy genes specifically in adult intestinal stem cells and quantified the populations of differentiated cells, progenitors and stem cells within the *Drosophila* posterior midgut. We also performed rescue experiments to identify the mechanisms regulated by the autophagy pathway in intestinal stem cells that lead to the disturbed organization of the tissue.

… adult *Drosophila* with dysfunctional autophagy in their intestinal stem cells have a shorter lifespan

**How would you explain the main findings of your paper to non-scientific family and friends?**

The gut can be seen as a tube made up of millions of specialized cells, each with a specific function. Most of these cells absorb nutrients, while a minority send signals to the brain to control the digestion. People with chronic IBDs, such as Crohn's disease, have many damaged cells in their intestines, which prevents the gut from functioning properly. Normally, these damaged cells should be replaced by new specialized cells produced by intestinal stem cells. Mutations in the mechanism that breaks down molecules inside cells, called autophagy, can increase the risk of developing chronic IBDs. We used adult *Drosophila* (fruit flies) to disrupt the autophagy mechanism in intestinal stem cells and counted the different specialized cell types of the gut. Our paper shows that even when the intestine is not damaged or inflamed, blocking autophagy in intestinal stem cells causes them to produce too many enteroendocrine cells – the cells that send signals to the brain. In the long term, adult *Drosophila* with dysfunctional autophagy in their intestinal stem cells have a shorter lifespan.

**What are the potential implications of these results for disease biology and the possible impact on patients?**

Therapies targeting JAK-STAT signalling have been explored, but they generally inhibit the kinase rather than the downstream transcription factor. Our data show that JAK-STAT signalling can be sustained by autophagy deficiency in intestinal stem cells, independently of kinase inhibition. Exploring this crosstalk more deeply could help us understand how inflammatory responses are maintained and why some therapies fail to reduce inflammation.

**Figure:**
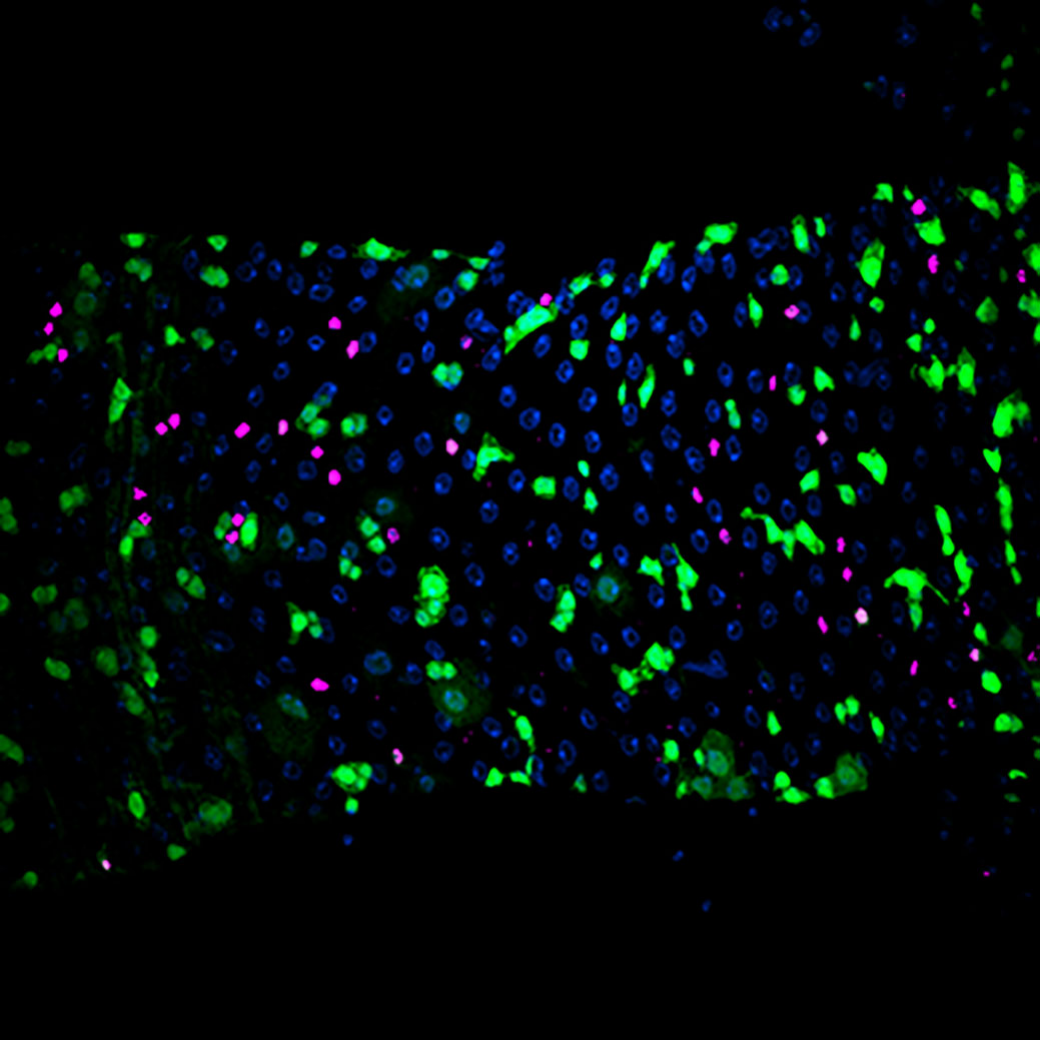
**An adult *Drosophila* intestine harbouring an overactivated JAK-STAT pathway (green) and a high number of enteroendocrine cells (magenta), after the inhibition of autophagy in intestinal stem cells.** Nuclei are labelled in blue.

**Why did you choose DMM for your paper?**

The use of non-mammalian models, such as *Drosophila*, helps uncover conserved mechanisms underlying disease. DMM publishes high-quality scientific articles and provides strong visibility for studies that utilize non-mammalian models.

**Given your current role, what challenges do you face and what changes could improve the professional lives of other scientists in this role?**

Trying to explore a mechanism in depth by following suggestions from the discussion of some papers can be very challenging, especially when experiments do not yield the expected results. When talking to other scientists at conferences, you realize that you are not alone in this struggle. Often, the authors of the publications you follow may have already tried similar approaches and failed, but you will never know unless you meet them personally. In research, we explore hypotheses and results can refute these hypotheses, meaning that negative results are just as important as positive ones. However, most published articles focus only on results that support the hypothesis. Publishing negative results could greatly benefit the scientific community, as it would help avoid long, repetitive experiments and save time and resources by showing that certain approaches or factors are not involved in a process especially if someone has already tested them.

Publishing negative results could greatly benefit the scientific community …

**What's next for you?**

I have just started my postdoc in a different field, neuroscience, taking on the challenge of working with a new model: neurons derived from induced pluripotent stem cells. Since I am interested in the gut-brain axis, I hope to gain sufficient experience and skills needed to combine this knowledge with my background in intestinal biology. I remain driven by the goal of becoming a principal investigator who studies the relationship between the intestine and the brain.

**Tell us something interesting about yourself that wouldn't be on your CV**

Besides being creative in science, I like to express my creativity by making amigurumi. These are cute, small stuffed toys handmade using crochet. I find it relaxing because you have to be one-hundred percent focused, which helps me stop thinking about upcoming experiments, ongoing projects...
